# Supplementary material for: n-Alkanes and n-Alkenes in Virgin Olive Oil from Calabria (South Italy): The Effects of Cultivar and Harvest Date
Source: Foods. 2021 Feb 1;10(2):290. doi: 10.3390/foods10020290 (PMC7912712; doi:10.3390/foods10020290)
Supplement: Supplementary file 1 [file foods-10-00290-s001.pdf]

Table S1. Heneicosane (C 21). Data are expressed as mg/kg and are given as mean ± SD (*n* = 8). Means and standard deviations were calculated on 8 replicates (4 replicates/year x two harvest years), in the harvest years 2016-2017 and 2017-2018. Means values in a vertical column with different capital letters and means in a line with small letters were significantly different according to the Tukey test at 5% probability.

Cultivar	3 Oct.	18 Oct.	3 Nov.	17 Nov.	5 Dec.	19 Dec.	3 Jan.	*Sign.*
**Cassanese**	0.10±0.01 abB	0.07±0.01 bC	0.13±0.01 aC	0.09±0.02 bC	0.13±0.04 aC	0.10±0.01 bB	0.10±0.01 abC	0.000
**Coratina**	0.73±0.39 aB	0.33±0.02 bcBC	0.33±0.03 bcBC	0.47±0.01 abC	0.14±0.01 cC	--	--	0.000
**Itrana**	0.18±0.08 aB	0.10±0.01 bBC	0.11±0.01 abC	0.11±0.03 bB	0.07±0.00 bC	--	--	0.002
**Leccino**	0.50±0.25 aB	0.38±0.02 abBC	0.52±0.03 aBC	0.48±0.05 aC	0.23±0.01 bC	--	--	0.001
**Nociara**	0.29±0.07 abB	0.44±0.14 abBC	0.53±0.18 aBC	0.40±0.19 abC	0.25±0.16 bC	0.26±0.00 bB	--	0.009
**Ottobratica**	0.32±0.04 aB	0.31±0.07 aBC	0.26±0.03 abC	0.19±0.03 bC	0.29±0.05 abC	0.26±0.11 abB	0.20±0.01 bB	0.001
**Pendolino**	0.58±0.27 abB	0.65±0.45 aB	0.25±0.06 bcC	0.18±0.04 cC	0.22±0.14 bcC	0.15±0.01 cB	--	0.001
**Picholine**	0.33±0.03 dB	0.65±0.14 cB	0.91±0.29 bB	1.24±0.08 aB	1.09±0.02 abB	--	--	0.000
**Sinopolese**	3.84±0.97 bA	4.59±0.77 abA	5.85±0.93 abA	6.67±2.10 aA	6.05±2.02 abA	4.88±1.20 abA	3.90±0.04 bA	0.003
Sign.	0.000	0.000	0.000	0.000	0.000	0.000	0.000	

Table S2. Docosane (C 22). Data are expressed as mg/kg and are given as mean ± SD (*n* = 8). Means and standard deviations were calculated on 8 replicates (4 replicates/year x two harvest years), in the harvest years 2016-2017 and 2017-2018. Means values in a vertical column with different capital letters and means in a line with small letters were significantly different according to the Tukey test at 5% probability.

**Cultivar**	**3 Oct.**	**18 Oct.**	**3 Nov.**	**17 Nov.**	**5 Dec.**	**19 Dec.**	**3 Jan.**	*Sign.*
**Cassanese**	0.24±0.01 abB	0.17±0.01 bcB	0.24±0.03 abC	0.16±0.02 cB	0.30±0.11 aB	0.23±0.01 bcB	0.17±0.01 bcC	0.000
**Coratina**	1.04±0.69 aB	0.35±0.03 bcB	0.43±0.09 bcC	0.77±0.01 abB	0.21±0.01 cB	--	--	0.001
**Itrana**	0.34±0.13 aB	0.23±0.01 bcB	0.25±0.02 abC	0.21±0.03 bcB	0.14±0.00 cB	--	--	0.000
**Leccino**	0.57±0.08 aB	0.48±0.10 abB	0.38±0.09 bcC	0.37±0.12 bcB	0.24±0.01 cB	--	--	0.000
**Nociara**	0.50±0.02 bcB	0.82±0.08 aB	0.93±0.13 aBC	0.75±0.22 abB	0.49±0.25 cB	0.50±0.01 bcB	--	0.000
**Ottobratica**	1.28±0.11 aB	0.99±0.20 aB	0.62±0.15 bC	0.36±0.02 bB	0.48±0.19 bB	0.49±0.31 bB	0.40±0.01 bB	0.000
**Pendolino**	0.35±0.03 aB	0.27±0.04 bB	0.24±0.03 bcC	0.22±0.02 cdB	0.19±0.01 deB	0.18±0.01 eB	--	0.000
**Picholine**	1.04±0.06 cB	1.31±0.17 bcB	1.67±0.46 abB	2.09±0.53 aB	1.69±0.02 abB	--	--	0.000
**Sinopolese**	13.35±2.51 abA	14.72±1.87 abA	15.33±1.26 aA	16.93±3.13 aA	16.83±5.13 aA	13.56±2.37 abA	10.33±0.04 bA	0.003
Sign.	0.000	0.000	0.000	0.000	0.000	0.000	0.000	

Table S3. Tricosene (C 23:1). Data are expressed as mg/kg and are given as mean ± SD (*n* = 8). Means and standard deviations were calculated on 8 replicates (4 replicates/year x two harvest years), in the harvest years 2016-2017 and 2017-2018. Means values in a vertical column with different capital letters and means in a line with small letters were significantly different according to the Tukey test at 5% probability.

**Cultivar**	**3 Oct.**	**18 Oct.**	**3 Nov.**	**17 Nov.**	**5 Dec.**	**19 Dec.**	**3 Jan.**	*Sign.*
**Cassanese**	0.03±0.01 cE	0.03±0.01 cB	0.05±0.01 aE	0.05±0.00 aC	0.03±0.01 bcD	0.05±0.01 abC	0.03±0.01 cC	0.000
**Coratina**	0.33±0.09 aD	0.21±0.08 abB	0.24±0.10 abD	0.15±0.00 bcC	0.09±0.01 cD	--	--	0.000
**Itrana**	0.04±0.01 abE	0.02±0.01 bB	0.06±0.02 aE	0.04±0.01 abC	0.05±0.00 aD	--	--	0.001
**Leccino**	0.06±0.02 bcE	0.06±0.01 bB	0.09±0.01 aDE	0.06±0.01 bcC	0.04±0.01 cD	--	--	0.000
**Nociara**	0.11±0.06 aE	0.09±0.06 aB	0.07±0.04 aE	0.10±0.07 aC	0.04±0.02 aD	0.03±0.00 aC	--	0.061
**Ottobratica**	0.91±0.20 aB	0.70±0.06 bA	0.44±0.07 cC	0.25±0.06 dBC	0.20±0.07 dC	0.23±0.07 dB	0.24±0.01 dB	0.000
**Pendolino**	0.10±0.03 aE	0.08±0.05 aB	0.02±0.01 bE	0.03±0.01 bC	0.03±0.02 bD	0.02±0.01 bC	--	0.000
**Picholine**	0.69±0.05 aC	0.78±0.16 aA	0.62±0.11 aB	0.44±0.08 bAB	0.44±0.02 bB	--	--	0.000
**Sinopolese**	1.24±0.06 aA	0.82±0.30 bA	0.84±0.20 bA	0.64±0.37 abA	0.64±0.10 abA	0.45±0.07 bA	0.58±0.04 abA	0.000
Sign.	0.000	0.000	0.000	0.000	0.000	0.000	0.000	

Table S4. Tricosane (C 23). Data are expressed as mg/kg and are given as mean ± SD (*n* = 8). Means and standard deviations were calculated on 8 replicates (4 replicates/year x two harvest years), in the harvest years 2016-2017 and 2017-2018. Means values in a vertical column with different capital letters and means in a line with small letters were significantly different according to the Tukey test at 5% probability.

Cultivar	3 Oct.	18 Oct.	3 Nov.	17 Nov.	5 Dec.	19 Dec.	3 Jan.	*Sign.*
**Cassanese**	1.05±0.03 aE	0.79±0.05 abcE	0.73±0.08 abcG	0.55±0.08 bcD	0.88±0.54 abC	0.48±0.03 cD	0.42±0.02 cC	0.000
**Coratina**	6.19±1.73 aCDE	4.69±1.18 aDE	4.68±0.77 aE	2.62±0.01 bCD	1.80±0.01 bC	--	--	0.000
**Itrana**	3.65±1.68 aCDE	1.83±0.15 bDE	1.62±0.55 bFG	0.84±0.10 bD	0.82±0.00 bC	--	--	0.000
**Leccino**	9.71±2.62 aC	6.99±3.42 abD	3.62±0.88 bcEF	3.33±1.19 cCD	2.79±0.01 cC	--	--	0.000
**Nociara**	8.71±0.45 bcCD	12.71±1.93 aC	12.04±2.82 abC	9.38±2.98 abC	5.40±3.07 cC	5.40±0.01 cB	--	0.000
**Ottobratica**	21.03±3.01 aB	15.81±2.41 bBC	7.91±1.66 cD	4.05±0.29 dCD	3.80±0.48 dC	3.32±2.03 dC	3.16±0.01 dB	0.000
**Pendolino**	2.99±0.16 aDE	2.54±0.35 bDE	1.76±0.20 cFG	1.41±0.03 dD	1.13±0.17 deC	0.90±0.01 eD	--	0.000
**Picholine**	19.79±1.44 aB	20.04±1.34 aB	18.93±1.64 abB	19.62±1.06 aB	17.05±0.03 bB	--	--	0.002
**Sinopolese**	85.94±9.06 aA	87.98±6.75 aA	90.48±1.27 aA	87.05±12.16 aA	86.26±13.71 aA	65.28±0.54 bA	60.03±0.08 bA	0.000
Sign.	0.000	0.000	0.000	0.000	0.000	0.000	0.000	

Table S5. Tetracosene (C 24:1). Data are expressed as mg/kg and are given as mean ± SD (*n* = 8). Means and standard deviations were calculated on 8 replicates (4 replicates/year x two harvest years), in the harvest years 2016-2017 and 2017-2018. Means values in a vertical column with different capital letters and means in a line with small letters were significantly different according to the Tukey test at 5% probability.

**Cultivar**	**3 Oct.**	**18 Oct.**	**3 Nov.**	**17 Nov.**	**5 Dec.**	**19 Dec.**	**3 Jan.**	*Sign.*
**Cassanese**	0.03±0.01 abD	0.02±0.01 bB	0.04±0.01 aC	0.01±0.00 bD	0.02±0.01 bD	0.02±0.00 bC	0.02±0.01 abC	0.000
**Coratina**	0.25±0.09 aC	0.16±0.05 abB	0.19±0.08 aC	0.16±0.01 abCD	0.07±0.01 bD	--	--	0.000
**Itrana**	0.02±0.00 aD	0.01±0.01 abB	0.02±0.00 abC	0.01±0.00 bD	0.00±0.00 cD	--	--	0.000
**Leccino**	0.02±0.01 aD	0.02±0.01 aB	0.02±0.00 aC	0.02±0.00 aD	0.01±0.00 aD	--	--	0.249
**Nociara**	0.09±0.01 aD	0.07±0.02 abB	0.06±0.01 bcC	0.05±0.01 bcdD	0.03±0.02 cD	0.04±0.00 cdC	--	0.000
**Ottobratica**	1.05±0.14 aA	0.84±0.04 bA	0.49±0.03 cB	0.27±0.06 dBC	0.19±0.07 dC	0.24±0.10 dB	0.24±0.01 dB	0.000
**Pendolino**	0.02±0.01 aD	0.02±0.01 aB	0.02±0.01 aC	0.02±0.01 aD	0.02±0.01 aD	0.01±0.00 aC	--	0.296
**Picholine**	0.79±0.08 aB	0.79±0.08 aA	0.62±0.09 bB	0.48±0.08 cAB	0.44±0.02 cB	--	--	0.000
**Sinopolese**	1.17±0.10 aA	0.78±0.28 bcA	0.98±0.35 abA	0.58±0.33 cA	0.65±0.11 bcA	0.43±0.06 cA	0.51±0.03 cA	0.000
Sign.	0.000	0.000	0.000	0.000	0.000	0.000	0.000	

Table S6. Tetracosane (C 24). Data are expressed as mg/kg and are given as mean ± SD (*n* = 8). Means and standard deviations were calculated on 8 replicates (4 replicates/year x two harvest years), in the harvest years 2016-2017 and 2017-2018. Means values in a vertical column with different capital letters and means in a line with small letters were significantly different according to the Tukey test at 5% probability.

Cultivar	3 Oct.	18 Oct.	3 Nov.	17 Nov.	5 Dec.	19 Dec.	3 Jan.	*Sign.*
**Cassanese**	1.53±0.19 aE	1.05±0.06 bF	0.88±0.10 bcF	0.60±0.09 dF	0.71±0.26 cdD	0.60±0.04 dC	0.47±0.03 dC	0.000
**Coratina**	4.98±1.42 aDE	3.65±0.73 abEF	3.72±0.83 abD	2.59±0.02 bcDE	1.75±0.01 cCD	--	--	0.000
**Itrana**	3.44±1.27 aDE	1.93±0.16 bF	1.68±0.65 bcEF	0.84±0.10 cF	0.91±0.01 bcD	--	--	0.000
**Leccino**	11.41±2.57 aC	7.88±4.88 aDE	3.20±0.89 bDE	2.73±1.41 bDE	2.86±0.01 bCD	--	--	0.000
**Nociara**	7.27±0.62 aCD	9.03±1.44 aCD	8.17±1.62 aC	6.70±1.72 aC	4.11±1.90 bC	4.13±0.01 bB	--	0.000
**Ottobratica**	16.40±1.88 aB	12.84±1.43 bBC	7.14±1.41 cC	3.97±0.33 dD	3.23±0.02 dCD	3.16±1.94 dB	2.77±0.01 dB	0.000
**Pendolino**	2.88±0.13 aE	2.15±0.09 bF	1.64±0.10 cEF	1.37±0.04 dEF	1.09±0.13 eCD	0.94±0.01 eC	--	0.000
**Picholine**	17.22±1.07 aB	16.24±0.57 aB	14.37±0.4 4 bB	13.94±0.74 bB	11.83±0.02 cB	--	--	0.000
**Sinopolese**	54.09±5.59 aA	53.10±4.91 abA	47.071.37 bcA	44.76±0.86 cA	45.87±4.49 cA	36.56±1.89 dA	33.05±0.04 dA	0.000
Sign.	0.000	0.000	0.000	0.000	0.000	0.000	0.000	

Table S7. Pentacosene (C 25:1). Data are expressed as mg/kg and are given as mean ± SD (*n* = 8). Means and standard deviations were calculated on 8 replicates (4 replicates/year x two harvest years), in the harvest years 2016-2017 and 2017-2018. Means values in a vertical column with different capital letters and means in a line with small letters were significantly different according to the Tukey test at 5% probability.

**Cultivar**	**3 Oct.**	**18 Oct.**	**3 Nov.**	**17 Nov.**	**5 Dec.**	**19 Dec.**	**3 Jan.**	*Sign.*
**Cassanese**	0.13±0.03 aDE	0.08±0.03 bcD	0.09±0.01 bcC	0.07±0.01 cBC	0.07±0.03 cCD	0.11±0.01 abcB	0.11±0.01 abC	0.000
**Coratina**	0.45±0.11 abC	0.39±0.15 abC	0.60±0.38 aB	0.28±0.02 abB	0.19±0.01 bC	--	--	0.010
**Itrana**	0.04±0.01 bE	0.04±0.02 bD	0.09±0.01 aC	0.05±0.01 bBC	0.07±0.00 aCD	--	--	0.000
**Leccino**	0.05±0.02 abDE	0.06±0.01 aD	0.05±0.03 abC	0.04±0.01 abC	0.02±0.00 bD	--	--	0.009
**Nociara**	0.13±0.06 abDE	0.14±0.02 aD	0.06±0.01 bcC	0.09±0.03 abcBC	0.07±0.06 bcCD	0.05±0.00 cB	--	0.001
**Ottobratica**	1.47±0.19 aA	1.31±0.04 aA	0.96±0.11 bA	0.57±0.08 cA	0.50±0.17 cB	0.58±0.13 cA	0.60±0.01 cB	0.000
**Pendolino**	0.27±0.25 aCD	0.11±0.09 abD	0.03±0.02 bC	0.03±0.01 bC	0.01±0.01 bD	0.02±0.01 bB	--	0.003
**Picholine**	1.03±0.06 abB	1.11±0.16 aAB	0.92±0.13 bcA	0.73±0.08 dA	0.79±0.02 cdA	--	--	0.000
**Sinopolese**	1.31±0.08 aA	0.94±0.22 bcB	1.00±0.28 abA	0.70±0.36 bcdA	0.74±0.11 bcdA	0.54±0.08 dA	0.62±0.02 cdA	0.000
Sign.	0.000	0.000	0.000	0.000	0.000	0.000	0.000	

Table S8. Pentacosane (C 25). Data are expressed as mg/kg and are given as mean ± SD (*n* = 8). Means and standard deviations were calculated on 8 replicates (4 replicates/year x two harvest years), in the harvest years 2016-2017 and 2017-2018. Means values in a vertical column with different capital letters and means in a line with small letters were significantly different according to the Tukey test at 5% probability.

**Cultivar**	**3 Oct.**	**18 Oct.**	**3 Nov.**	**17 Nov.**	**5 Dec.**	**19 Dec.**	**3 Jan.**	*Sign.*
**Cassanese**	7.00±1.42 aE	4.80±0.45 bD	3.78±0.41 bcG	2.71±0.37 cdH	2.40±0.30 dE	2.92±0.21 cdC	2.43±0.16 dC	0.000
**Coratina**	11.10±1.17 aE	9.69±0.78 aCD	10.09±1.82 aDE	6.94±0.02 bE	6.61±0.01 bD	--	--	0.000
**Itrana**	7.20±0.70 aE	5.30±0.02 bD	4.79±0.91 bFG	2.92±0.17 cGH	3.16±0.01 cE	--	--	0.000
**Leccino**	17.70±3.40 aD	13.15±6.03 aC	7.05±0.86 bEF	6.43±1.55 bEF	6.45±0.01 bD	--	--	0.000
**Nociara**	11.46±0.85 aE	12.63±1.56 aC	11.23±1.87 aD	9.94±1.82 abD	7.15±2.31 bD	7.61±0.01 bBC	--	0.000
**Ottobratica**	34.15±3.43 aB	29.34±2.65 bB	19.90±3.03 cC	13.17±0.33 dC	11.99±1.05 dC	11.20±4.51 dB	11.40±0.02 dB	0.000
**Pendolino**	9.26±0.15 aE	7.32±0.44 bCD	5.69±0.72 cFG	4.73±0.23 dFG	4.01±0.34 eE	3.51±0.01 eC	--	0.000
**Picholine**	27.95±2.11 aC	29.09±0.60 bB	23.31±0.37 cB	22.55±0.36 cdB	21.05±0.02 dB	--	--	0.000
**Sinopolese**	91.35±6.78 aA	86.50±8.46 aA	73.68±2.53 bA	68.79±2.01 bcA	72.31±1.54 bA	61.24±6.25 cdA	59.63±0.05 dA	0.000
Sign.	0.000	0.000	0.000	0.000	0.000	0.000	0.000	

Table S9. Hexacosane (C 26). Data are expressed as mg/kg and are given as mean ± SD (*n* = 8). Means and standard deviations were calculated on 8 replicates (4 replicates/year x two harvest years), in the harvest years 2016-2017 and 2017-2018. Means values in a vertical column with different capital letters and means in a line with small letters were significantly different according to the Tukey test at 5% probability.

Cultivar	**3 Oct.**	**18 Oct.**	**3 Nov.**	**17 Nov.**	**5 Dec.**	**19 Dec.**	**3 Jan.**	*Sign.*
**Cassanese**	2.14±0.35 aDE	1.57±0.19 bCD	1.29±0.14 bcF	1.05±0.10 cdC	0.88±0.11 dG	1.11±0.09 cdC	0.91±0.07 dC	0.000
**Coratina**	3.18±0.99 aC	2.20±0.04 bcC	2.23±0.34 bcD	2.63±0.02 abB	1.67±0.01 cD	--	--	0.000
**Itrana**	1.55±0.11 aE	1.39±0.11 bD	1.28±0.01 bF	1.06±0.02 cC	0.97±0.01 cG	--	--	0.000
**Leccino**	2.94±0.50 aC	2.25±0.93 abC	1.43±0.04 bcF	1.26±0.24 cC	1.35±0.01 cEF	--	--	0.000
**Nociara**	1.85±0.03 aE	1.74±0.06 aCD	1.55±0.10 bEF	1.43±0.09 bcC	1.24±0.13 dF	1.33±0.00 cdC	--	0.000
**Ottobratica**	4.32±0.25 aB	3.71±0.17 abB	3.27±0.24 bcB	2.74±0.12 cdB	2.71±0.21 cdB	2.74±0.80 cdB	2.53±0.01 dB	0.000
**Pendolino**	2.77±0.08 aCD	2.29±0.23 bC	1.82±0.10 cE	1.66±0.05 cdC	1.50±0.09 dDE	1.48±0.01 dC	--	0.000
**Picholine**	3.30±0.08 aC	3.08±0.14 abB	2.82±0.23 bcC	2.73±0.32 cdB	2.44±0.02 dC	--	--	0.000
**Sinopolese**	8.75±0.33 aA	7.51±0.57 bA	5.85±0.14 cA	5.38±0.92 cA	5.83±0.27 cA	5.23±0.74 cA	5.15±0.04 cA	0.000
Sign.	0.000	0.000	0.000	0.000	0.000	0.000	0.000	

Table S10. Eptacosane (C 27). Data are expressed as mg/kg and are given as mean ± SD (*n* = 8). Means and standard deviations were calculated on 8 replicates (4 replicates/year x two harvest years), in the harvest years 2016-2017 and 2017-2018. Means values in a vertical column with different capital letters and means in a line with small letters were significantly different according to the Tukey test at 5% probability.

Cultivar	3 Oct.	18 Oct.	3 Nov.	17 Nov.	5 Dec.	19 Dec.	3 Jan.	*Sign.*
**Cassanese**	6.71±1.09 aDE	5.05±0.32 bE	4.36±0.38 bcC	3.62±0.69 cE	3.42±0.36 cE	4.68±0.34 bD	4.82±0.32 bC	0.000
**Coratina**	10.12±0.02 aCD	9.81±0.09 aC	9.92±2.07 aB	7.77±0.01 bC	6.64±0.00 bC	--	--	0.000
**Itrana**	5.43±1.34 aE	5.40±0.60 aE	4.99±1.22 abC	4.64±0.96 abDE	3.66±0.01 bE	--	--	0.022
**Leccino**	11.85±3.62 aC	7.84±1.19 bD	6.23±0.81 bC	6.45±0.98 bCD	5.53±0.01 bCD	--	--	0.000
**Nociara**	6.92±0.34 aDE	5.49±0.06 bE	4.95±0.16 cC	4.60±0.09 dDE	4.57±0.24 dDE	4.79±0.01 cdD	--	0.000
**Ottobratica**	17.14±1.76 aB	13.60±0.8 9 cdB	15.05±0.03 abcA	14.50±1.98 abcdA	16.36±1.8 1 abA	12.00±2.14 dB	13.78±0.02 bcdB	0.000
**Pendolino**	17.33±0.14 aB	14.37±1.6 5 bB	11.42±1.57 cB	10.20±0.22 cdB	8.84±0.56 dB	8.96±0.01 dC	--	0.000
**Picholine**	6.94±0.91 aDE	6.59±1.06 aDE	6.60±2.13 aC	6.26±1.81 aCD	5.13±0.02 aD	--	--	0.233
**Sinopolese**	24.46±3.21 aA	19.11±0.1 8 bA	17.22±2.09 bcA	15.39±1.83 cA	17.36±0.5 1 bcA	18.61±0.53 bA	19.98±0.03 bA	0.000
Sign.	0.000	0.000	0.000	0.000	0.000	0.000	0.000	

Table S11. Octacosane (C 28). Data are expressed as mg/kg and are given as mean ± SD (n = 8). Means and standard deviations were calculated on 8 replicates (4 replicates/year x two harvest years), in the harvest years 2016-2017 and 2017-2018. Means values in a vertical column with different capital letters and means in a line with small letters were significantly different according to the Tukey test at 5% probability.

Cultivar	3 Oct.	18 Oct.	3 Nov.	17 Nov.	5 Dec.	19 Dec.	3 Jan.	*Sign.*
**Cassanese**	1.54±0.18 aC	1.03±0.18 bD	0.86±0.08 bcD	0.70±0.09 cdC	0.60±0.09 dD	0.83±0.07 bcB	0.74±0.06 cdC	0.000
**Coratina**	2.96±0.91 aB	2.04±0.09 bC	1.94±0.55 bcC	2.49±0.01 abA	1.20±0.01 cC	--	--	0.000
**Itrana**	0.71±0.03 aD	0.84±0.11 aD	0.82±0.14 aD	0.74±0.14 aC	0.53±0.00 bD	--	-	0.000
**Leccino**	1.76±0.36 aC	1.22±0.35 bD	0.93±0.06 bD	0.91±0.07 bC	0.94±0.01 bCD	--	--	0.000
**Nociara**	1.67±0.12 aC	1.19±0.14 bD	1.07±0.06 bcD	0.98±0.03 cC	0.96±0.04 cCD	1.00±0.00 cB	--	0.000
**Ottobratica**	3.59±0.43 aAB	2.70±0.22 bB	2.65±0.13 bA	2.64±0.29 bA	2.66±0.37 bA	2.39±0.77 bA	2.34±0.02 bA	0.000
**Pendolino**	4.05±0.23 aA	3.36±0.51 bA	2.60±0.03 cAB	2.44±0.05 cA	2.17±0.24 cB	2.39±0.01 cA	--	0.000
**Picholine**	1.10±0.13 aCD	0.95±0.10 abD	0.89±0.16 abcD	0.81±0.24 bcC	0.65±0.02 cD	--	--	0.000
**Sinopolese**	3.95±0.65 aA	2.81±0.09 bB	2.25±0.05 bcBC	1.84±0.55 cB	2.03±0.60 bcB	1.95±0.63 cA	2.21±0.03 bcB	0.000
Sign.	0.000	0.000	0.000	0.000	0.000	0.000	0.000	

Table S12. Nonacosane (C 29). Data are expressed as mg/kg and are given as mean ± SD (*n* = 8). Means and standard deviations were calculated on 8 replicates (4 replicates/year x two harvest years), in the harvest years 2016-2017 and 2017-2018. Means values in a vertical column with different capital letters and means in a line with small letters were significantly different according to the Tukey test at 5% probability.

**Cultivar**	**3 Oct.**	**18 Oct.**	**3 Nov.**	**17 Nov.**	**5 Dec.**	**19 Dec.**	**3 Jan.**	*Sign.*
**Cassanese**	7.28±1.66 aC	4.51±0.90 bF	3.45±0.39 bcdC	2.79±0.30 cdE	2.35±0.49 dE	3.70±0.28 bcdB	3.96±0.26 bcC	0.000
**Coratina**	12.38±1.30 abB	13.43±1.13 aC	13.28±5.23 aB	8.46±0.02 bcC	6.28±0.01 cC	--	--	0.000
**Itrana**	4.95±0.67 aC	4.96±0.17 aF	4.30±0.85 abC	3.71±0.45 bcDE	3.31±0.01 cDE	--	--	0.000
**Leccino**	10.72±1.58 aB	7.55±0.42 bDE	5.89±0.29 cC	5.99±0.35 cD	5.19±0.01 cCD	--	--	0.000
**Nociara**	11.71±2.03 aB	8.15±1.66 bD	6.55±1.03 bcC	6.05±0.94 bcD	5.81±0.49 cCD	6.10±0.01 bcB	--	0.000
**Ottobratica**	23.99±1.80 aA	17.19±0.19 bB	18.23±0.55 bA	16.32±2.03 bA	17.87±2.88 bA	11.69±2.32 cA	12.59±0.02 cA	0.000
**Pendolino**	25.15±0.55 aA	20.24±3.14 bA	15.35±2.01 cAB	12.98±0.35 cdB	11.49±1.24 dB	11.85±0.01 dA	--	0.000
**Picholine**	6.86±1.06 aC	5.15±0.48 bEF	4.52±1.18 bcC	3.92±1.23 bcDE	3.27±0.02 cDE	--	--	0.000
**Sinopolese**	24.45±3.33 aA	16.38±0.59 bB	13.91±1.77 bcB	9.92±2.63 cC	9.84±2.52 cB	9.78±3.32 cA	12.05±0.04 cB	0.000
Sign.	0.000	0.000	0.000	0.000	0.000	0.000	0.000	

Table S13. Triacontane (C 30). Data are expressed as mg/kg and are given as mean ± SD (*n* = 8). Means and standard deviations were calculated on 8 replicates (4 replicates/year x two harvest years), in the harvest years 2016-2017 and 2017-2018. Means values in a vertical column with different capital letters and means in a line with small letters were significantly different according to the Tukey test at 5% probability.

**Cultivar**	**3 Oct.**	**18 Oct.**	**3 Nov.**	**17 Nov.**	**5 Dec.**	**19 Dec.**	**3 Jan.**	*Sign.*
**Cassanese**	1.02±0.12 aCD	0.58±0.12 bEF	0.50±0.05 bcB	0.39±0.03 cdD	0.33±0.07 dC	0.51±0.03 bcB	0.54±0.04 bC	0.000
**Coratina**	2.61±0.65 aAB	1.89±0.24 abAB	1.74±0.73 bA	2.14±0.01 abA	0.82±0.01 cB	--	--	0.000
**Itrana**	0.38±0.01 aE	0.36±0.01 abF	0.33±0.04 bcB	0.30±0.00 cD	0.25±0.00 dC	--	--	0.000
**Leccino**	1.30±0.18 aC	0.95±0.02 bDEF	0.81±0.12 bcB	0.77±0.05 cC	0.69±0.01 cB	--	--	0.000
**Nociara**	1.44±0.26 aC	1.04±0.33 abCDE	0.92±0.29 bB	0.84±0.24 bC	0.70±0.16 bB	0.79±0.01 bB	--	0.000
**Ottobratica**	2.24±0.16 aB	1.66±0.07 bcBC	1.78±0.04 abA	1.69±0.14 bcB	1.72±0.28 bA	1.44±0.61 bcA	1.22±0.01 cA	0.000
**Pendolino**	2.88±0.51 aA	2.48±0.84 aA	1.64±0.07 bA	1.48±0.13 bB	1.44±0.42 bA	1.50±0.01 bA	--	0.000
**Picholine**	0.71±0.03 aDE	0.53±0.03 bEF	0.46±0.04 bcB	0.40±0.09 cdD	0.33±0.02 dC	--	--	0.000
**Sinopolese**	2.05±0.11 aB	1.46±0.26 abBCD	1.76±0.79 aA	0.89±0.18 bcC	0.81±0.15 cB	0.80±0.24 cB	0.98±0.07 bcB	0.000
Sign.	0.000	0.000	0.000	0.000	0.000	0.000	0.000	

Table S14. Entriacontane (C 31). Data are expressed as mg/kg and are given as mean ± SD (*n* = 8). Means and standard deviations were calculated on 8 replicates (4 replicates/year x two harvest years), in the harvest years 2016-2017 and 2017-2018. Means values in a vertical column with different capital letters and means in a line with small letters were significantly different according to the Tukey test at 5% probability.

Cultivar	3 Oct.	18 Oct.	3 Nov.	17 Nov.	5 Dec.	19 Dec.	3 Jan.	*Sign.*
**Cassanese**	3.93±1.12 aDE	1.96±0.65 bcE	1.34±0.19 cdC	1.17±0.24 cdEF	0.88±0.26 dF	1.70±0.12 cdB	2.78±0.18 bC	0.000
**Coratina**	6.39±0.40 abBC	7.14±1.38 abA	7.71±3.70 aA	4.52±0.02 bcB	3.07±0.01 cBC	--	--	0.001
**Itrana**	1.67±0.03 aF	1.41±0.11 bE	1.14±0.08 cC	1.02±0.19 cF	1.33±0.00 bDEF	--	--	0.000
**Leccino**	4.97±0.32 aCD	3.58±0.37 bCD	2.85±0.39 cBC	3.08±0.62 bcC	2.46±0.01 cCD	--	--	0.000
**Nociara**	4.36±1.02 aDE	2.74±0.56 bDE	2.09±0.37 bC	1.96±0.34 bDE	1.86±0.33 bDEF	2.10±0.00 bB	--	0.000
**Ottobratica**	8.65±0.79 aA	5.70±0.13 bAB	6.35±0.29 bA	5.68±0.65 bA	6.54±1.49 bA	3.93±1.31 cA	3.96±0.01 cA	0.000
**Pendolino**	9.63±2.20 aA	6.66±1.91 bA	5.16±0.55 bcAB	3.94±0.79 cB	3.81±0.90 cB	4.05±0.01 cA	--	0.000
**Picholine**	2.57±0.21 aEF	1.92±0.05 bE	1.55±0.09 cC	1.31±0.28 cdEF	1.27±0.01 dEF	--	--	0.000
**Sinopolese**	7.95±1.42 aAB	4.64±0.14 bcBC	5.30±2.30 bAB	2.77±0.41 cdCD	2.35±0.31 dCDE	2.60±0.78 dB	3.21±0.04 cdB	0.000
Sign.	0.000	0.000	0.000	0.000	0.000	0.000	0.000	

Table S15. Dotriacontane (C 32). Data are expressed as mg/kg and are given as mean ± SD (*n* = 8). Means and standard deviations were calculated on 8 replicates (4 replicates/year x two harvest years), in the harvest years 2016-2017 and 2017-2018. Means values in a vertical column with different capital letters and means in a line with small letters were significantly different according to the Tukey test at 5% probability.

**Cultivar**	**3 Oct.**	**18 Oct.**	**3 Nov.**	**17 Nov.**	**5 Dec.**	**19 Dec.**	**3 Jan.**	*Sign.*
**Cassanese**	0.45±0.02 aC	0.21±0.06 cdDE	0.18±0.02 cdeB	0.16±0.04 deE	0.12±0.02 eD	0.22±0.02 cB	0.37±0.03 bA	0.000
**Coratina**	0.41±0.05 bcCD	0.53±0.11 bA	0.60±0.27 bA	1.27±0.02 aA	0.29±0.00 cABC	--	--	0.000
**Itrana**	0.14±0.01 aE	0.11±0.01 bcE	0.11±0.01 cB	0.09±0.02 dF	0.12±0.00 bD	--	--	0.000
**Leccino**	0.38±0.07 aCD	0.24±0.03 bDE	0.23±0.03 bB	0.24±0.05 bD	0.27±0.01 bBCD	--	--	0.000
**Nociara**	0.37±0.02 aCD	0.21±0.05 bDE	0.16±0.01 cB	0.14±0.01 cEF	0.16±0.04 cCD	0.16±0.00 cB	--	0.000
**Ottobratica**	0.49±0.09 aBC	0.32±0.02 aCD	0.38±0.03 aAB	0.41±0.01 aB	0.43±0.08 aA	0.51±0.36 aA	0.27±0.01 aB	0.055
**Pendolino**	0.68±0.19 aA	0.46±0.15 abAB	0.37±0.02 bAB	0.33±0.03 bC	0.41±0.21 bAB	0.31±0.01 bAB	--	0.001
**Picholine**	0.25±0.03 aDE	0.19±0.03 bDE	0.18±0.03 bB	0.14±0.03 cEF	0.14±0.01 cCD	--	--	0.000
**Sinopolese**	0.63±0.14 aAB	0.38±0.05 abBC	0.63±0.43 aA	0.23±0.03 bD	0.23±0.06 bCD	0.22±0.05 bB	0.27±0.04 bB	0.000
Sign.	0.000	0.000	0.000	0.000	0.000	0.009	0.000	

Table S16. Tritriacontane (C 33). Data are expressed as mg/kg and are given as mean ± SD (*n* = 8). Means and standard deviations were calculated on 8 replicates (4 replicates/year x two harvest years), in the harvest years 2016-2017 and 2017-2018. Means values in a vertical column with different capital letters and means in a line with small letters were significantly different according to the Tukey test at 5% probability.

**Cultivar**	**3 Oct.**	**18 Oct.**	**3 Nov.**	**17 Nov.**	**5 Dec.**	**19 Dec.**	**3 Jan.**	*Sign.*
**Cassanese**	1.44±0.37 aBCD	0.68±0.22 bE	0.43±0.03 bcD	0.48±0.16 bcCDE	0.35±0.08 cD	0.73±0.05 bABC	1.67±0.11 aA	0.000
**Coratina**	1.89±0.77 aBC	1.58±0.22 abAB	1.96±0.86 aAB	1.53±0.01 abA	0.86±0.01 bB	--	--	0.010
**Itrana**	0.56±0.01 aD	0.43±0.01 bE	0.30±0.04 cD	0.32±0.12 cE	0.44±0.00 bCD	--	--	0.000
**Leccino**	1.16±0.02 aCD	0.66±0.02 bE	0.54±0.09 bCD	0.63±0.21 bCD	0.59±0.01 bBCD	--	--	0.000
**Nociara**	1.25±0.22 aCD	0.76±0.09 bDE	0.47±0.05 cD	0.44±0.02 cDE	0.48±0.07 cCD	0.51±0.01 cC	--	0.000
**Ottobratica**	1.86±0.43 aBC	1.09±0.07 bCD	1.51±0.03 abABC	1.53±0.03 abA	1.81±0.39 aA	1.13±0.62 bA	0.99±0.02 bC	0.000
**Pendolino**	2.38±1.10 aAB	1.20±0.52 bBC	1.13±0.34 bBCD	0.70±0.16 bC	0.70±0.19 bBC	0.57±0.01 bBC	--	0.000
**Picholine**	0.74±0.13 aD	0.46±0.07 bE	0.49±0.13 bCD	0.27±0.04 cE	0.30±0.02 cD	--	--	0.000
**Sinopolese**	3.02±0.51 aA	1.72±0.05 bcA	2.39±1.35 abA	1.11±0.13 cB	0.85±0.12 cB	1.03±0.13 cAB	1.14±0.05 cB	0.000
Sign.	0.000	0.000	0.000	0.000	0.000	0.002	0.000	

Table S17. Tetratriacontane (C 34). Data are expressed as mg/kg and are given as mean ± SD (*n* = 8). Means and standard deviations were calculated on 8 replicates (4 replicates/year x two harvest years), in the harvest years 2016-2017 and 2017-2018. Means values in a vertical column with different capital letters and means in a line with small letters were significantly different according to the Tukey test at 5% probability.

Cultivar	3 Oct.	18 Oct.	3 Nov.	17 Nov.	5 Dec.	19 Dec.	3 Jan.	*Sign.*
**Cassanese**	0.18±0.06 aABC	0.06±0.02 bCD	0.05±0.01 bB	0.06±0.02 bBC	0.05±0.01 bCD	0.10±0.01 bAB	0.21±0.02 aA	0.000
**Coratina**	0.20±0.10 aAB	0.12±0.01 bAB	0.15±0.05 abB	0.07±0.01 bBC	0.09±0.00 bBCD	--	--	0.001
**Itrana**	0.05±0.01 aE	0.04±0.00 abD	0.03±0.00 bcB	0.03±0.01 cC	0.05±0.00 aCD	--	--	0.000
**Leccino**	0.08±0.01 bCDE	0.05±0.01 cCD	0.05±0.01 cB	0.06±0.01 cBC	0.12±0.01 aABC	--	--	0.000
**Nociara**	0.12±0.03 aBCDE	0.06±0.01 bCD	0.05±0.01 bB	0.04±0.01 bC	0.05±0.02 bCD	0.06±0.00 bB	--	0.000
**Ottobratica**	0.16±0.05 abABCD	0.10±0.02 bAB	0.14±0.02 abB	0.15±0.04 abA	0.17±0.02 abA	0.27±0.23 aA	0.08±0.01 bC	0.030
**Pendolino**	0.16±0.07 aABCD	0.09±0.03 abBC	0.09±0.02 abB	0.08±0.01 abBC	0.15±0.11 aAB	0.04±0.01 bB	--	0.010
**Picholine**	0.06±0.02 aDE	0.05±0.01 aCD	0.05±0.01 aB	0.04±0.01 aC	0.03±0.01 aD	--	--	0.021
**Sinopolese**	0.25±0.06 abA	0.13±0.04 bA	0.35±0.28 aA	0.10±0.07 bAB	0.12±0.05 bABC	0.08±0.04 bB	0.11±0.03 bB	0.003
Sign.	0.000	0.000	0.000	0.000	0.000	0.006	0.000	

Table S18. Pentatriacontane (C 35). Data are expressed as mg/kg and are given as mean ± SD (*n* = 8). Means and standard deviations were calculated on 8 replicates (4 replicates/year x two harvest years), in the harvest years 2016-2017 and 2017-2018. Means values in a vertical column with different capital letters and means in a line with small letters were significantly different according to the Tukey test at 5% probability.

**Cultivar**	**3 Oct.**	**18 Oct.**	**3 Nov.**	**17 Nov.**	**5 Dec.**	**19 Dec.**	**3 Jan.**	*Sign.*
**Cassanese**	0.33±0.03 bCD	0.18±0.04 dDEF	0.11±0.01 dC	0.16±0.07 dBCD	0.12±0.03 dCD	0.25±0.02 cAB	0.60±0.04 aA	0.000
**Coratina**	0.30±0.06 aCDEF	0.28±0.01 aBC	0.18±0.14 abBC	0.15±0.01 bCD	0.23±0.01 abBC	--	--	0.004
**Itrana**	0.14±0.05 aEF	0.09±0.01 bF	0.09±0.02 bC	0.09±0.04 bDE	0.12±0.00 abCD	--	--	0.015
**Leccino**	0.32±0.02 aCDE	0.15±0.03 cEF	0.14±0.01 cBC	0.15±0.04 cCD	0.22±0.00 bBC	--	--	0.000
**Nociara**	0.26±0.03 aDEF	0.20±0.02 bCDE	0.11±0.01 cC	0.10±0.01 cCDE	0.13±0.01 cCD	0.12±0.00 cB	--	0.000
**Ottobratica**	0.47±0.09 abBC	0.29±0.02 bcB	0.44±0.03 abAB	0.51±0.07 aA	0.54±0.06 aA	0.42±0.25 abA	0.22±0.01 cB	0.000
**Pendolino**	0.54±0.25 aAB	0.24±0.10 bBCD	0.27±0.13 bBC	0.17±0.04 bBC	0.30±0.16 abB	0.10±0.01 bB	--	0.000
**Picholine**	0.12±0.02 aF	0.10±0.02 abF	0.07±0.02 bcC	0.05±0.02 cE	0.06±0.02 cD	--	--	0.000
**Sinopolese**	0.69±0.06 aA	0.44±0.08 abcA	0.62±0.46 abA	0.24±0.03 cB	0.23±0.05 cBC	0.29±0.07 bcAB	0.25±0.03 cB	0.000
Sign.	0.000	0.000	0.000	0.000	0.000	0.000	0.000	

Table S19. Total alkanes. Data are expressed as mg/kg and are given as mean ± SD (n = 8). Means and standard deviations were calculated on 8 replicates (4 replicates/year x two harvest years), in the harvest years 2016-2017 and 2017-2018. Means values in a vertical column with different capital letters and means in a line with small letters were significantly different according to the Tukey test at 5% probability.

Cultivar	3 Oct.	18 Oct.	3 Nov.	17 Nov.	5 Dec.	19 Dec.	3 Jan.	*Sign.*
**Cassanese**	34.97±6.46 aF	22.71±3.02 bE	18.32±1.89 bcE	14.70±1.46 cE	13.54±1.36 cE	18.16±1.34 bcD	20.17±1.34 bC	0.000
**Coratina**	64.47±7.93 aDE	57.73±5.75 abD	58.97±16.97 abC	44.41±0.03 bcC	31.65±0.03 cCD	--	--	0.000
**Itrana**	30.40±1.63 aF	24.42±0.52 bE	21.84±0.02 cDE	16.90±1.09 dE	15.87±0.01 dDE	--	--	0.000
**Leccino**	75.37±14.75 aCDE	53.36±16.96 bD	33.85±1.07 cD	32.88±2.21 cD	29.92±0.01 cCDE	--	--	0.000
**Nociara**	58.18±5.91 aE	57.21±8.13 aD	50.81±8.70 abC	43.76±8.66 bcC	33.36±9.21 cC	34.88±0.01 cC	--	0.000
**Ottobratica**	136.08±14.26 aB	105.66±6.14 bB	85.61±7.32 cB	67.91±4.19 cdB	70.60±7.90 cdB	54.95±18.32 dB	55.92±0.03 dB	0.000
**Pendolino**	81.61±5.20 aCD	64.30±10.26 bCD	49.43±4.97 cC	41.92±1.08 cdCD	37.44±4.89 dC	36.94±0.02 dC	--	0.000
**Picholine**	88.99±2.55 aC	83.34±4.76 abC	76.83±6.90 bB	75.36±6.80 bB	66.33±0.05 cB	--	--	0.000
**Sinopolese**	324.76±15.83 aA	301.46±24.43 abA	282.67±6.32 bcA	262.08±9.91 cA	266.98±22.76 cA	222.11±10.51 dA	212.28±0.07 dA	0.000
Sign.	0.000	0.000	0.000	0.000	0.000	0.000	0.000	

Table S20. Total alkenes. Data are expressed as mg/kg and are given as mean ± SD (*n* = 8). Means and standard deviations were calculated on 8 replicates (4 replicates/year x two harvest years), in the harvest years 2016-2017 and 2017-2018. Means values in a vertical column with different capital letters and means in a line with small letters were significantly different according to the Tukey test at 5% probability.

Cultivar	3 Oct.	18 Oct.	3 Nov.	17 Nov.	5 Dec.	19 Dec.	3 Jan.	*Sign.*
**Cassanese**	0.19±0.03 aD	0.13±0.04 bC	0.18±0.03 abD	0.14±0.02 bD	0.12±0.03 bDE	0.17±0.03 abC	0.16±0.02 abC	0.002
**Coratina**	1.03±0.29 aC	0.77±0.27 bcB	1.03±0.56 aC	0.59±0.03 bcCD	0.35±0.03 cD	--	--	0.003
**Itrana**	0.10±0.02 dD	0.08±0.03 dC	0.16±0.02 aD	0.10±0.01 dD	0.13±0.01 bDE	--	--	0.000
**Leccino**	0.13±0.04 aD	0.14±0.01 aC	0.16±0.03 aD	0.12±0.02 aD	0.08±0.01 bE	--	--	0.000
**Nociara**	0.32±0.12 aD	0.29±0.09 abBC	0.19±0.06 abcD	0.24±0.10 abcD	0.14±0.10 bcDE	0.12±0.01 cC		0.002
**Ottobratica**	3.42±0.53 aA	2.84±0.13 bA	1.89±0.20 cB	1.09±0.20 dBC	0.90±0.32 dC	1.05±0.3 dB	1.08±0.03 dB	0.000
**Pendolino**	0.39±0.28 aD	0.20±0.15 abBC	0.07±0.04 bD	0.08±0.02 bD	0.06±0.04 bE	0.06±0.02 bC	--	0.001
**Picholine**	2.51±0.20 abB	2.68±0.39 aA	2.17±0.34 bB	1.64±0.24 cAB	1.67±0.05 cB	--	--	0.000
**Sinopolese**	3.74±0.10 aA	2.54±0.78 bcA	2.83±0.82 abA	1.92±1.05 bcdA	2.03±0.27 bcdA	1.42±0.15 dA	1.71±0.07 cdA	0.000
Sign.	0.000	0.000	0.000	0.000	0.000	0.000	0.000	

Table S21. Alkanes/Alkenes ratio. Data are given as mean ± SD (*n* = 8). Means and standard deviations were calculated on 8 replicates (4 replicates/year x two harvest years), in the harvest years 2016-2017 and 2017-2018. Means values in a vertical column with different capital letters and means in a line with small letters were significantly different according to the Tukey test at 5% probability.

Cultivar	3 Oct.	18 Oct.	3 Nov.	17 Nov.	5 Dec.	19 Dec.	3 Jan.	*Sign.*
**Cassanese**	181.28±14.57 aBC	188.86±42.66 aBC	108.95±30.12 bB	107.69±6.92 bCDE	112.19±24.87 bB	107.89±8.57 bC	126.83±10.12 bA	0.000
**Coratina**	65.12±10.39 bD	81.70±21.49 abBC	65.83±19.28 bB	75.32±3.48 abDE	90.15±6.72 aB	--	--	0.023
**Itrana**	309.74±37.97 aB	353.41±132.55 aAB	136.76±13.59 bB	169.66±17.56 bBCD	121.72±6.19 bB	--	--	0.000
**Leccino**	602.90±157.11 aA	368.42±96.27 bcAB	220.75±33.61 cB	279.35±61.20 bcB	395.99±34.37 bB	--	--	0.000
**Nociara**	199.31±55.66 bBC	204.05±32.49 abBC	282.11±48.44 abB	198.51±49.22 bBC	366.52±207.81 aB	285.84±12.87 abB	--	0.021
**Ottobratica**	40.01±2.09 cD	37.27±3.73 cC	46.12±8.75 cB	63.52±7.66 bDE	84.69±21.25 aB	52.02±2.69 bcC	51.78±1.37 bcB	0.000
**Pendolino**	360.24±270.21 aB	579.06±449.51 aA	926.37±630.51 aA	564.43±144.97 aA	950.96±568.29 aA	693.34±193.62 aA	--	0.148
**Picholine**	35.58±1.92 abD	31.92±6.44 bC	36.64±8.92 abB	47.18±10.87 aE	39.81±1.35 abB	--	--	0.012
**Sinopolese**	86.91±3.48 bD	126.34±29.46 abBC	106.73±28.09 abB	178.36±93.40 aBCD	132.61±9.53 abB	158.38±23.28 abBC	124.12±5.05 abA	0.008
Sign.	0.000	0.000	0.000	0.000	0.000	0.000	0.000	

Table S22. Total odd-chain alkanes. Data are expressed as mg/kg and are given as mean ± SD (*n* = 8), Means and standard deviations were calculated on 8 replicates (4 replicates/year x two harvest years), in the harvest years 2016-2017 and 2017-2018. Means values in a vertical column with different capital letters and means in a line with small letters were significantly different according to the Tukey test at 5% probability.

Cultivar	3 Oct.	18 Oct.	3 Nov.	17 Nov.	5 Dec.	19 Dec.	3 Jan.	*Sign.*
**Cassanese**	27.86±5.69 aE	18.04±2.44 bE	14.32±1.47 bcG	11.58±1.18 cE	10.55±1.06 cE	14.56±1.07 bcD	16.77±1.09 bC	0.000
**Coratina**	49.09±3.22 aD	46.95±4.59 aCD	48.16±14.30 aCD	32.45±0.01 bCD	25.63±0.02 bC	--	--	0.000
**Itrana**	23.78±0.38 aE	19.52±0.47 bE	17.34±0.45 cFG	13.63±1.02 dE	12.90±0.00 dDE	--	--	0.000
**Leccino**	56.92±11.16 aCD	40.29±10.75 bD	26.83±0.15 cEF	26.54±0.48 cD	23.46±0.01 cCD	--	--	0.000
**Nociara**	44.97±4.97 aD	43.12±6.02 aD	37.97±6.50 abDE	32.87±6.37 bcCD	25.65±6.68 cC	26.91±0.04 cC		0.000
**Ottobratica**	107.60±11.33 aB	83.34±4.50 bB	69.64±5.61 bcB	55.96±4.02 cdeB	59.19±7.15 cdB	43.95±13.28 eB	46.31±0.01 deB	0.000
**Pendolino**	67.86±4.25 aC	53.21±8.55 bCD	41.02±5.06 cD	34.33±0.96 cdC	30.50±3.70 dC	30.09±0.04 dC	--	0.000
**Picholine**	65.31±1.52 aC	60.99±3.76 abC	56.38±5.59 bC	55.21±4.87 bcB	49.22±0.08 cB	--	--	0.000
**Sinopolese**	241.70±8.32 aA	221.35±16.82 bA	209.44±5.09 bcA	191.94±7.54 cA	195.25±14.09 cA	163.71±9.35 dA	160.18±0.08 dA	0.000
Sign.	0.000	0.000	0.000	0.000	0.000	0.000	0.000	

Table S23. Total even-chain alkanes. Data are expressed as mg/kg and are given as mean ± SD (*n* = 8), Means and standard deviations were calculated on 8 replicates (4 replicates/year x two harvest years), in the harvest years 2016-2017 and 2017-2018. Means values in a vertical column with different capital letters and means in a line with small letters were significantly different according to the Tukey test at 5% probability.

Cultivar	3 Oct.	18 Oct.	3 Nov.	17 Nov.	5 Dec.	19 Dec.	3 Jan.	*Sign.*
**Cassanese**	7.12±0.78 aEF	4.67±0.58 bD	4.00±0.42 bcH	3.12±0.28 dE	2.99±0.34 dD	3.60±0.27 cdD	3.41±0.25 cdC	0.000
**Coratina**	15.38±4.71 aD	10.79±1.16 bCD	10.81±2.67 bDE	11.96±0.03 abC	6.02±0.01 cCD	--	--	0.000
**Itrana**	6.62±1.25 aF	4.90±0.05 bD	4.49±0.44 bGH	3.26±0.08 cE	2.97±0.01 cD	--	--	0.000
**Leccino**	18.44±3.59 aCD	13.07±6.21 aC	7.01±0.92 bFG	6.34±1.73 bD	6.47±0.02 bCD	--	--	0.000
**Nociara**	13.21±0.94 aDE	14.09±2.11 aC	12.84±2.20 aD	10.89±2.29 abC	7.71±2.53 bCD	7.97±0.04 bBC		0.000
**Ottobratica**	28.47±2.94 aB	22.32±1.64 bB	15.97±1.72 cC	11.94±0.17 cdC	11.41±0.75 dBC	11.00±5.03 dB	9.61±0.03 dB	0.000
**Pendolino**	13.75±0.96 aD	11.09±1.71 bCD	8.41±0.12 cEF	7.60±0.13 cD	6.94±1.19 cCD	6.85±0.02 cCD	--	0.000
**Picholine**	23.68±1.04 aBC	22.34±1.01 abB	20.46±1.31 bcB	20.15±1.94 cB	17.11±0.03 dB	--	--	0.000
**Sinopolese**	83.06±7.51 aA	80.11±7.61 abA	73.23±1.23 bcA	70.13±2.39 cA	71.73±8.68 bcA	58.41±1.18 dA	52.10±0.15 dA	0.000
Sign.	0.000	0.000	0.000	0.000	0.000	0.000	0.000	
